# A Comparative Analysis of Generalized Peritonitis Secondary to Upper Gastrointestinal Perforation: Surgical Approaches, Antibiotic Management, and Patient Outcomes

**DOI:** 10.7759/cureus.102483

**Published:** 2026-01-28

**Authors:** Alfayina Rodriguez Abreu, Alekcei Evgenevich Klimov, Nikolay Vyacheslavovich Lebedev, Ian Wooley, Arianna Trujillo Polier, Isy Cedeño Carpio, Yoelina Vargas Nuñez, Daniela Alexandra Sierra Guzman, Andreina Rosario Rosario

**Affiliations:** 1 General Surgery, City Clinical Hospital Named After V.V. Vinogradov, Moscow, RUS; 2 Surgery, Peoples' Friendship University of Russia, Moscow, RUS; 3 Medicine, Universidad Iberoamericana (UNIBE), Santo Domingo, DOM; 4 General Surgery, Hospital Materno Infantil San Lorenzo de Los Mina, Santo Domingo, DOM; 5 Medicine, Servicio Nacional de Salud (SNS) Cibao Northwest, Valverde, DOM; 6 Medicine, Autonomous University of Santo Domingo (UASD), Santo Domingo, DOM

**Keywords:** gastric perforation, generalized peritonitis, laparoscopic repair, perforated peptic ulcer, time-to-surgery

## Abstract

Generalized peritonitis due to gastric or peptic ulcer perforation (PULP) carries substantial morbidity and mortality. This study is a Preferred Reporting Items for Systematic Reviews and Meta-Analyses-guided systematic review, conducted using PubMed, MEDLINE, Embase, Scopus, and Cochrane, from January 2020 to August 2025. Adults with generalized peritonitis from gastric/PULP reporting surgery, antibiotics, and outcomes were included. Two independent reviewers conducted the screening and data extraction, assessing the risk of bias (RoB) using the Newcastle-Ottawa Scale for observational studies and RoB2 for randomized controlled trials. A narrative synthesis was conducted due to clinical/methodological heterogeneity. Twenty-five studies met the criteria. The contemporary series showed variable 30-day or in-hospital mortality. The highest rates were observed when the time to surgery exceeded 12-24 hours, in cases of shock, among older patients, and for those with an American Society of Anesthesiologists classification of ≥III. Laparoscopic repair, when feasible, was consistently associated with shorter length of stay and fewer wound infections than open repair, with similar mortality; conversion was more likely with higher PULP scores. Omental patch repair predominated and showed lower complications than gastric resection, the latter reserved for large, malignant, or recurrent ulcers. Empiric broad-spectrum antibiotics were universally initiated, with variable stewardship/deescalation. Outcomes hinge on early resuscitation, rapid source control, and structured sepsis care. Laparoscopy reduces morbidity when expertise/resources are available; globally, the omental patch remains first-line. Standardized antibiotic pathways and risk-based triage may reduce variation, but timely access and critical care capacity are decisive.

## Introduction and background

Generalized peritonitis remains a significant clinical challenge, particularly when it is secondary to gastric perforation, which can arise from various underlying pathologies. This condition represents a surgical emergency and is associated with a high mortality rate if not promptly recognized and managed. The severity of generalized peritonitis results from the widespread inflammation of the peritoneum, triggered by the leakage of gastric contents into the abdominal cavity, causing a cascade of events that culminate in sepsis and multiorgan failure if left untreated [1]. Understanding the etiology, clinical presentation, and the urgency of intervention in gastric perforation-induced peritonitis is crucial for improving patient outcomes.

The stomach is a vital organ responsible for the initial phases of digestion, including the breakdown of food by gastric acid and enzymes. The gastric mucosa, which protects the stomach from its acidic environment, may be compromised by various factors, leading to perforation and subsequent peritonitis. A perforation disrupts the integrity of the gastric wall, allowing the highly acidic gastric contents to escape into the sterile peritoneal cavity. This escape triggers an intense inflammatory response, often resulting in generalized peritonitis, which can rapidly progress to sepsis and organ dysfunction [2].

The pathophysiological sequence of events begins with the leakage of gastric acid, digestive enzymes, and bacterial flora into the peritoneal space. This results in chemical irritation and a subsequent inflammatory response. The bacteria from the gastrointestinal tract, such as *Escherichia coli* and *Bacteroides fragilis*, are potent instigators of infection and sepsis. Additionally, digestive enzymes, especially pepsin and bile salts, exacerbate tissue injury, leading to necrosis and inflammation. The peritoneum, initially sterile, becomes a breeding ground for bacteria, leading to the systemic inflammatory response syndrome and, if untreated, progressing to septic shock [3].

Several factors contribute to the occurrence of gastric perforation. Among the most common causes are peptic ulcers, which are responsible for a significant proportion of gastric perforations, especially in older patients [4]. Chronic exposure to gastric acid in peptic ulcer disease weakens the mucosal lining of the stomach, creating ulcerations that can deepen over time, ultimately leading to perforation. The increasing prevalence of *Helicobacter pylori* infection and the widespread use of nonsteroidal anti-inflammatory drugs have further contributed to the rise in peptic ulcer-related perforations [5].

Another significant cause of gastric perforation is trauma, which can result from blunt abdominal injuries or penetrating wounds. Trauma-related perforation is often seen in high-velocity accidents, where the sheer force causes a rupture of the stomach wall. Iatrogenic perforations, although less common, can occur during endoscopic procedures or gastric surgeries, particularly in patients with preexisting gastric diseases [6]. Rarely, gastric tumors such as adenocarcinoma or gastrointestinal stromal tumors may erode through the stomach wall, causing perforation and subsequent peritonitis [7].

Infectious diseases such as tuberculosis can also lead to gastric perforation, although this is uncommon in developed countries. In regions with a high incidence of gastrointestinal tuberculosis, however, perforation is a recognized complication. Moreover, in patients with underlying malignancies or immunosuppressed individuals, spontaneous perforations have been documented, albeit infrequently [8].

The clinical presentation of generalized peritonitis due to gastric perforation is typically dramatic, with patients presenting acutely ill. The hallmark symptom is sudden-onset, severe abdominal pain, which is often described as "sharp" or "burning" in nature. This pain is usually generalized, affecting the entire abdomen, and is exacerbated by movement. Accompanying symptoms include nausea, vomiting, and signs of shock such as hypotension and tachycardia, which suggest the development of sepsis [9].

Physical examination may reveal signs consistent with peritonitis, including abdominal distension, rigidity, and rebound tenderness. Patients often exhibit a "board-like" abdomen, reflecting the intense muscular guarding that occurs in response to peritoneal irritation. The presence of free air under the diaphragm, often visible on upright chest X-rays, is a classic radiographic sign of gastric perforation and indicates that air has escaped from the gastrointestinal tract into the peritoneal cavity [10].

The diagnosis of generalized peritonitis secondary to gastric perforation relies on a combination of clinical suspicion, imaging studies, and laboratory investigations. Early diagnosis is crucial to prevent the progression of sepsis and multiple organ failure. While a detailed history and physical examination are the first steps, imaging plays a pivotal role in confirming the diagnosis. An upright chest X-ray or abdominal radiograph often reveals free intraperitoneal air, a key indicator of perforation. In more complex cases or where there is diagnostic uncertainty, a computed tomography (CT) scan of the abdomen may be warranted, providing more detailed information regarding the location and extent of the perforation [11].

Laboratory investigations typically show evidence of an acute inflammatory response, including leukocytosis, elevated C-reactive protein, and metabolic acidosis. In patients who are hemodynamically unstable, blood cultures may reveal bacterial growth, confirming the presence of sepsis [12].

Once the diagnosis of gastric perforation with generalized peritonitis is established, prompt surgical intervention is essential. The primary goals of treatment are to repair the perforation, remove contaminated material from the peritoneal cavity, and provide adequate resuscitation to reverse shock and prevent multiorgan failure. Early antibiotic therapy targeting gastrointestinal flora is initiated to combat sepsis, alongside aggressive fluid resuscitation to support circulation [13].

The prognosis for patients with generalized peritonitis secondary to gastric perforation depends on several factors, including the timeliness of intervention, the patient’s underlying health status, and the severity of the peritonitis. Delays in treatment are associated with significantly higher mortality rates, emphasizing the importance of early recognition and rapid surgical management [14].

## Review

Study design

This literature review focuses on generalized peritonitis caused by gastric perforation. The study adheres to a systematic review framework, following the Preferred Reporting Items for Systematic Reviews and Meta-Analyses guidelines. The review includes peer-reviewed articles, clinical studies, case reports, and randomized controlled trials on gastric perforation and subsequent generalized peritonitis. The data extraction process involved a rigorous assessment of inclusion and exclusion criteria, quality appraisal, and data synthesis to ensure comprehensive coverage of the topic.

Literature search strategy

A comprehensive and reproducible literature search was conducted to identify relevant studies addressing generalized peritonitis secondary to gastric or peptic ulcer perforation (PULP). The search was performed across PubMed/MEDLINE, Scopus, Embase, and the Cochrane Library, covering the period from January 2020 to August 2025.

The search strategy was developed a priori and combined controlled vocabulary (Medical Subject Headings, where applicable) with free-text terms related to gastric perforation, peritonitis, emergency surgery, and sepsis. Boolean operators (“AND,” “OR”) were used to link concepts, and database-specific syntax was applied as required. Searches were limited to human studies, adult populations (≥18 years), and articles published in English or with an available English translation.

Inclusion and exclusion criteria

The inclusion criteria comprised clinical studies including prospective and retrospective designs, cohort analyses, RCTs, and case series published between January 2020 and August 2025. Eligible populations were adult patients (≥18 years) with generalized peritonitis secondary to gastric perforation (primary focus), PULPs (gastric and/or duodenal), or upper gastrointestinal perforations in which gastric perforation was analyzed as a subgroup. Studies were required to report surgical management (open or laparoscopic) and/or perioperative care such as antibiotics and sepsis management. Outcomes of interest included at least one of the following: mortality, complication rates, length of hospital stay, antibiotic regimens, diagnostic methods, or timing of surgery. Only peer-reviewed articles published in English or with available English translations and indexed in PubMed, Scopus, Embase, Web of Science, or the Cochrane Library were considered.

The exclusion criteria encompassed studies limited exclusively to pediatric patients (<18 years), unless adults were included without a separate pediatric analysis. Research focusing solely on peritonitis from non-upper gastrointestinal sources, such as appendicitis, diverticulitis, trauma to the small or large bowel, or gynecologic perforations, was excluded unless gastric or PULPs were part of the cohort. Abstracts, editorials, letters to the editor, expert opinions lacking primary data, and conference proceedings without complete datasets were not considered. Additionally, duplicate publications from overlapping patient cohorts were excluded, with only the most comprehensive or updated version retained. Finally, non-human studies, including animal models and laboratory-based research, were not eligible.

Data extraction

A standardized extraction of abbreviations was established a priori and used consistently throughout the manuscript: American Society of Anesthesiologists (ASA) physical status classification, PULP score, length of stay (LOS), and intensive care unit (ICU). Postoperative morbidity was recorded using the Clavien-Dindo classification, and major complications were defined as Clavien-Dindo grade ≥ II when reported. A sheet was employed to ensure uniformity across studies. The following variables were recorded: 1) study characteristics: author(s), year of publication, and study design (retrospective, prospective, or observational); 2) patient population: total sample size, mean age, and sex distribution as reported in each study; 3) diagnostic modalities: utilization rates of preoperative CT scans, clinical diagnosis, and ultrasound as reported across series; 4) timing of surgical intervention: proportion of patients undergoing surgery within six hours of diagnosis vs. delayed interventions beyond 12 hours; 5) misdiagnosis rates: frequency of cases initially misclassified or identified late in the course of evaluation; and 6) operative details and outcomes: type of surgical procedure performed, postoperative complications, length of hospital stay, and mortality.

The primary outcome was 30-day all-cause mortality (or in-hospital mortality when 30-day was unavailable). Secondary outcomes included complications (Clavien-Dindo ≥ II), sepsis and septic shock per Sepsis-3, wound infection, intra-abdominal abscess, leak, ICU admission and LOS, hospital LOS, reoperation, and 30-day readmission. When studies employed alternative definitions, we extracted verbatim text and noted discrepancies in the footnotes.

Sepsis and septic shock were extracted using Sepsis-3 definitions when explicitly reported. When included studies applied alternative sepsis definitions, we extracted the authors’ original terminology verbatim and flagged the definition as non-uniform in the evidence tables/footnotes to avoid inadvertent reclassification.

We extracted the empirical antibiotic spectrum, time-to-first dose, culture-guided deescalation, and total duration after source control. Where reported, we noted antifungal use among high-risk hosts and adherence to institutional stewardship.

Quality assessment

The quality of the included studies was assessed using the Newcastle-Ottawa Scale (NOS) for observational studies and the Cochrane Risk of Bias (RoB) tool for randomized trials. The NOS evaluates three main domains: selection of study groups, comparability of groups, and ascertainment of the exposure or outcome of interest. Studies scoring six to nine stars were considered high quality, whereas those scoring below six stars were categorized as low quality. For randomized trials, the Cochrane RoB tool evaluated factors such as random sequence generation, allocation concealment, blinding of participants and outcome assessors, and completeness of outcome data. Studies with low or unclear bias in key areas were deemed high quality.

Statistical analysis

Given the heterogeneity in study designs, patient populations, and outcome measures, a narrative synthesis was conducted. The results of the selected studies were summarized, and trends were identified in terms of patient demographics, causes of gastric perforation, surgical approaches, and clinical outcomes. Descriptive statistics were used to report means and medians for continuous variables such as hospital stay duration and age at diagnosis. Categorical variables, such as the type of surgery or the incidence of complications, were reported as percentages.

Ethical considerations

As this study is a literature review involving no direct patient contact or intervention, institutional review board approval was not required. All included data were derived from peer-reviewed sources and handled in accordance with principles of research integrity.

Overview of included studies

**Figure 1:**
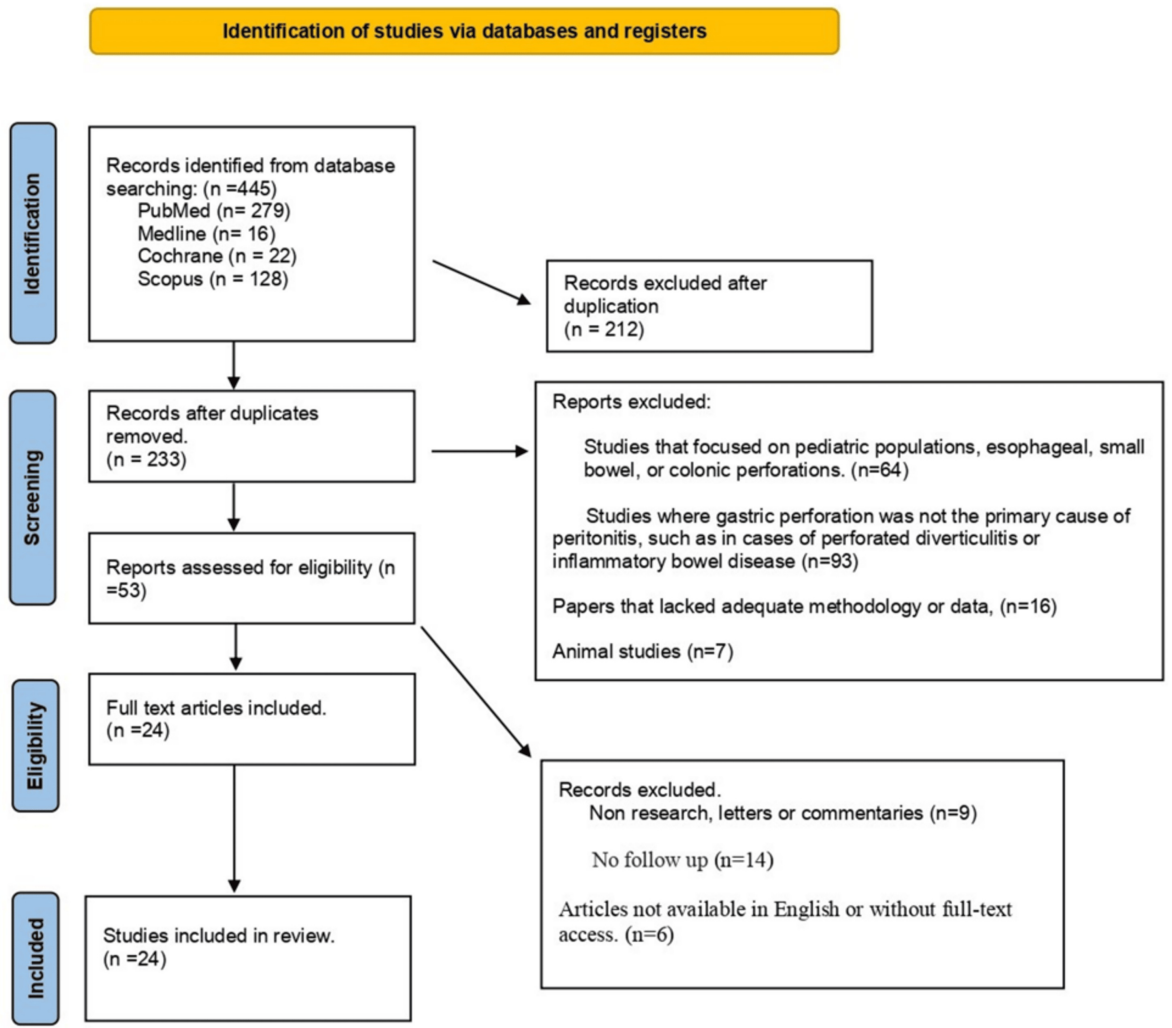
PRISMA flow diagram, illustrating literature selection and review processes PRISMA: Preferred Reporting Items for Systematic Reviews and Meta-Analyses

The initial database search yielded 445 articles from PubMed, MEDLINE, Scopus, Embase, and the Cochrane Library. After removing 212 duplicates, 233 articles remained for title and abstract screening. Of these, 233 were excluded as they did not meet the inclusion criteria, leaving 54 full-text articles for detailed assessment. After a thorough review, 25 studies were finally included in the systematic review (Figure 1, Table 1).

**Table 1:** Comparative results of generalized peritonitis (focus on gastric/PPU where available): mortality, complications, length of stay, surgical intervention, and sepsis/septic shock ^†^Statistical significance (p < 0.05) as reported in the original studies ASA: American Society of Anesthesiologists; GP: generalized peritonitis; IStiS: interrupted stitches; IQR: interquartile range; KnotS: knotless; LOS: length of stay; PPU: perforated peptic ulcer; PUD: peptic ulcer disease; PULP: peptic ulcer perforation; SSI: surgical site infection; TSA: trial sequential analysis

Study	Sample size	Mortality (%)	Complication (%)	Length of stay (days)	Surgical intervention (%)	Sepsis/septic shock (%)
Abouelazayem et al. [13]	1,874	9.3 (30-day)	48.5	Median: 7 days	80 open, remainder laparoscopic /other	Not reported explicitly^†^
Olausson et al. [14]	591 (124 GP)	18.5 (GP, 30-day)	87.1 (any, GP)	Median: 9.7 days (GP)	100 (major emergency surgery cohort)	ICU admission 39.5
Yamamoto et al. [15]	1,189 (derivation+validation)	7.6 (derivation), 6.8 (validation)	29-32 (Clavien ≥II)	Median: 12 days	100 (PPU surgery cohort)	Not reported
Sung et al. [16]	170 patients (gastrointestinal perforation requiring surgery; subset included gastric/PULPs)	6.4% (in-hospital)	41.8% (Clavien-Dindo ≥II; wound infection, intra-abdominal abscess, pneumonia most frequent)	Median: 11 days (IQR: 7-18)	94.7% emergency surgery (laparotomy or laparoscopy, depending on stability and contamination)	22.4% presented with or developed septic shock
Bejiga et al. [17]	140	12.9% (in-hospital)	43.5% (surgical site infection, sepsis, pneumonia most common)	Median: 10 days (range: 7-16)	100% (all underwent laparotomy with omental patch or definitive repair)	19.4% developed sepsis/septic shock
Jayaraman et al. [18]	Total 3,475 patients (open group: 3,135; laparoscopic: 340)	Open: 5.9%, laparoscopic: 3.8%	Bleeding: open 14.6% vs. laparoscopic 8.0%, pneumonia: Open 8.7% vs. laparoscopic 4.5%	Laparoscopic: 8.2; open: 9.4	100% of included patients underwent surgical repair (either open or laparoscopic) by definition of the cohort	Septic shock present in Open group ~10.5%, in Laparoscopic group ~4.4%n
Sohn et al. [19]	45 patients with PPU and generalized peritonitis	11% (in-hospital)	~36% (mainly intra-abdominal abscess, wound infection, pneumonia)	Median: 10 days (IQR: 7-15)	100% (all underwent emergency surgery; majority omental patch, minority resection)	(%): 18% developed sepsis, 7% progressed to septic shock
Ahmed et al. [20]	102 patients	11.8% (30-day mortality)	37.3% (postoperative complications, mainly wound infection, sepsis)	~9 days (range: 4-18)	100% (all underwent emergency laparotomy with repair)	Advanced age, delayed presentation (>24 hours), and shock at admission were strongly associated with poor outcomes
Soni et al. [21]	148 patients (74 laparoscopic, 74 open after matching)	~6.8% in laparoscopic group vs. ~10.8% in open group	Lower in the laparoscopic group (Clavien-Dindo ≥II ~24% vs. 38% in the open group)	7 laparoscopic vs. 11 open	100 op	12 laparoscopic vs. 19 open (sepsis); 4 vs. 7-8 (shock)
Koranne et al. [22]	60 patients	20% (12 out of 60 patients)	~35% (mainly wound infections, sepsis, pneumonia)	~10 days	100% (all underwent laparotomy with Graham’s omental patch or definitive surgery)	~18% developed sepsis; septic shock in ~8%-10% of cases (correlated strongly with higher scores)
Endeshaw et al. [23]	3,876	12.8-day pooled	35	9 pooled mean	100 surgery (pooled)	18 pooled
Mahlefahlo et al. [24]	75 patients with PPU	17.3% (13/75, in-hospital)	41% (mainly surgical site infection, intra-abdominal sepsis, pneumonia)	Mean: 12 days (range: 7-20)	100% (all underwent emergency laparotomy with Graham’s patch or resection)	Sepsis 22%; septic shock 9%
Murmu et al. [25]	104	14.4	38.5% overall (surgical site infection, intra-abdominal abscess, pneumonia most frequent)	Mean: 10 days (range: 6-18)	100	19.2 sepsis; 7.7 shock. Delayed presentation (>24 hours) and shock at admission were the strongest predictors of poor outcome
Dadfar and Edna [26]	404	16.2	~35% (postoperative morbidity; pneumonia and sepsis most frequent)	Mean: 9 days (declined from 12 days in early decades to ~7 days in later years)	100	12 sepsis; 6 shock; decline in incidence over time (likely due to Helicobacter pylori eradication and PPU use). Median age shifted upward (more elderly patients affected in recent decades). Mortality remained high, especially in elderly and delayed presenters
Hoshi et al. [27]	1,653	7.8	28% (Clavien-Dindo ≥II; pneumonia, SSI, intra-abdominal abscess common)	Median: 12 days (IQR: 8-18)	100% (laparotomy 72%, laparoscopy 28%)	14 sepsis; 6 shock; increasing trend toward laparoscopic repair (especially after 2017). Older age and high ASA class strongly associated with mortality. Time-to-surgery >12 hours significantly worsened outcomes
Wang et al. [28]	168	5.4	27	Mean: 8.2 days	100 (68 lap; 18% converted)	9 sepsis; 3 shock, higher PULP scores were strongly associated with conversion from laparoscopy to open repair. Patients with PULP ≥7 had significantly worse outcomes (mortality 18%). Suggested PULP as a dual prognostic tool (mortality + technical feasibility of laparoscopy)
Zheng et al. [29]	89,477 hospitalizations for PUD (subset with perforation analyzed separately) (National, China; perforation subset)	7.5% in perforated PUD subgroup	~30% (sepsis, bleeding, wound infection common)	Median: 10 days (IQR: 7-15)	100% for perforation cases (laparotomy most common; laparoscopy <15%)	12% developed sepsis; 5% progressed to septic shock. Elderly patients and those with comorbidities (diabetes, cardiovascular disease) had markedly higher mortality. Declining incidence of PUD overall, but perforated ulcer cases remained a major surgical burden. Mortality is higher in rural vs. urban hospitals, reflecting disparities in access and resources
Zhu et al. [30]	2,315 patients (1,725 omental patch vs. 590 gastric resection	Omental patch: 10%; gastric resection: 18%	Omental patch: 29%. Gastric resection: 38%. Complication risk is significantly lower with patch repair	Median: 11 days (patch) vs. 15 days (resection)	100	~15% across both groups, with no significant difference. Omental patch repair demonstrated better short-term outcomes (lower mortality and complications, shorter LOS). Gastric resection is reserved for large, malignant, or recurrent ulcers. Authors conclude that omental patch remains the procedure of choice in most settings, with gastric resection selectively indicated
Burale et al. [31]	232	12.9% (30-day)	41.4% (SSI, intra-abdominal abscess, pneumonia most common)	Median: 11 days (IQR: 8-16)	100% (all underwent laparotomy; Graham’s patch repair 95%)	18% developed sepsis; 7% presented with septic shock. Delayed presentation (>24 hours) and ASA ≥III were independent predictors of mortality. Smoking, comorbidities (diabetes, hypertension), and age >60 are strongly associated with poor outcomes. Early surgery (<12 hours) reduced mortality significantly
Sokhal et al. [32]	1,226	No significant difference between laparoscopic and open repair (both ~5%-7%)	Laparoscopic: significantly lower surgical site infections (OR ~0.45). No significant difference in intra-abdominal abscess or leak	Reduced by ~2 days in the laparoscopic arm (weighted mean difference -2.1 days)	Laparoscopy feasible in majority; conversion rates 10%-15%)	No statistically significant difference (laparoscopic vs. open ~11%-13%). TSA confirmed sufficient evidence that laparoscopy reduces wound infections and LOS. Mortality differences remain inconclusive due to low event rates. Authors conclude that laparoscopic repair should be considered the standard of care where surgical expertise and resources exist
An et al. [33]	144	15.3	37% (most commonly surgical site infection and intra-abdominal abscess)	Median: 12 days (IQR: 8-18)	100% (all underwent laparotomy, Graham’s patch mainstay)	21 sepsis; 9 shock. Independent predictors of mortality: delay to surgery >24 hours, ASA ≥III, and age >60 years. HIV-positive patients (17% of the cohort) had higher complication rates but not significantly higher mortality. Authors highlight the critical need for earlier presentation and access to surgery in Malawi
Costa et al. [34]	265 patients underwent laparoscopic PPU repair: 198 with interrupted stitches, 67 with knotless barbed suture	In propensity-matched groups: IStiS: 10.7%; knotless (KnotS): 7.1%	Postoperative morbidity (any) in matched groups: IStiS: 24.0%; KnotS: 32.7%	Mean: 10.5 ± 3.2 days	All patients in this cohort underwent laparoscopic PPU repair (i.e., 100% surgical intervention by laparoscopy)	Sepsis was reported in 5%
Gavriilidis et al. [35]	1,573 patients	~6.8% (no significant difference between laparoscopic omental patch, open omental patch, Graham’s patch, fibrin glue, or jejunal serosal patch)	28%-35% (lowest in the laparoscopic omental patch group)	Median ~8–10 days across interventions; laparoscopic approaches slightly shorter.	100% (all patients had operative intervention, varied by technique)	~10%-15%, no significant difference between groups. Laparoscopic omental patch had lower overall complications and slightly reduced hospital stay compared to open repair. No alternative method (falciform ligament, fibrin glue, jejunal serosal patch) demonstrated clear superiority in mortality outcomes
Kim et al. [36]	241	1.4 laparoscopic vs. 5.4 open	17.6 laparoscopic vs. 36.5 open	Laparoscopic: median 8 days; Open: median 12 days	100% (all underwent surgery, either laparoscopic or open)	Reported in 6.8% laparoscopic vs. 12.2% open (not statistically significant)

This review clarifies how time to surgery, operative strategy, and antimicrobial stewardship interact to shape outcomes in generalized peritonitis from gastric/PULP, while also highlighting system-level constraints that drive variability across settings.

Mortality and Drivers of Risk

Across contemporary series, 30-day or in-hospital mortality generally ranges from approximately 5% to 20%, with lower rates in high-resource systems and higher rates when presentation is delayed and critical-care capacity is limited [13,14,17,23,25,26]. Time-to-knife is consistently decisive: cohorts show sharp mortality escalation when repair is delayed beyond 12-24 hours, particularly in patients presenting with shock or advanced physiologic derangement [14,27,31]. Age and comorbidity burden (ASA ≥ III) further amplify risk across national and institutional datasets, reinforcing the need for early escalation to organ support in frail patients [26,27,33].

Laparoscopic vs. open repair

Comparative studies evaluating laparoscopic vs. open repair in generalized peritonitis secondary to upper gastrointestinal perforation consistently suggest lower wound-related complications and shorter length of hospital stay with a laparoscopic approach. However, these advantages are not uniform across all patient groups and must be interpreted in light of patient selection and disease severity. The most robust benefit signals are observed in hemodynamically stable patients with limited peritoneal contamination, where laparoscopy can be performed without delaying definitive source control [34,35].

Importantly, several included studies combine heterogeneous emergency pathologies or lack comprehensive adjustment for physiological status and contamination burden, limiting direct causal inference regarding equivalence in mortality between approaches. While most contemporary series report no significant mortality difference, this finding likely reflects appropriate selection rather than intrinsic equivalence of techniques [36,37].

Conversion from laparoscopy to open surgery is consistently associated with higher PULP scores, advanced physiological derangement, and gross peritoneal soiling, underscoring that conversion is primarily a marker of disease severity rather than technical failure. In such settings, prompt conversion is appropriate and may mitigate further delays in source control [32,34].

Taken together, these findings indicate that laparoscopy offers morbidity and recovery advantages in selected patients, whereas open surgery remains the preferred strategy in unstable patients or in the presence of extensive contamination. Surgical approach should therefore be individualized, with priority given to timely and effective source control over operative modality [38].

Procedure selection was individualized according to patient stability, physiological reserve, ulcer location, radiologic findings, and intraoperative conditions, with the primary objective of achieving rapid and effective source control while minimizing operative time in septic or unstable patients. Gastric resection was reserved for selected cases in which local factors or ulcer characteristics precluded safe patch repair [17,20,39,40]. Outcome-based comparisons between patch repair and gastric resection are discussed exclusively in the “Extent of Resection and Technique Selection” section.

Risk Stratification and Decision Support

Scores can provide guidance, but should not delay source control. The PULP score has dual utility: prognostication and prediction of conversion from laparoscopy; however, acute care teams caution against overreliance in time-sensitive settings, as clinical trajectory and rapid OR access remain paramount [16,27,28,41].

Early empirical broad-spectrum antibiotic coverage targeting gram-negative and anaerobic organisms is nearly universal across the included studies, consistent with international guidelines recommending prompt initiation, with subsequent culture-guided deescalation [13,16,39,42-49]. No single regimen demonstrated superiority, and observed variation reflects local resistance patterns, microbiology access, and resource availability; aminoglycoside-based combinations remain common in low- and middle-income country (LMIC) settings due to cost and access considerations [50-53]. Following effective source control, shorter courses (approximately three to five days) are favored in many high-income cohorts, whereas prolonged therapy (7-10 days) persists in late presenters or heavily contaminated cases [39,54]. Persistently high rates of sepsis and septic shock (often 15%-25%) emphasize that antibiotics cannot compensate for delayed surgery or inadequate resuscitation, underscoring the need to integrate antimicrobial stewardship with timely operative intervention and bundled sepsis care [13,16,22,40].

System-level disparities

The reviewed literature demonstrates a persistent divergence in outcomes between high-income countries (HICs) and LMICs in cases of generalized peritonitis caused by upper gastrointestinal perforation. Reports from well-resourced health systems consistently describe lower mortality and complication rates, frequently below 10%, a finding attributed to earlier diagnosis, reliable access to CT, broader implementation of minimally invasive surgery, and the availability of structured postoperative critical care [13,22,36,41]. In contrast, mortality rates reported in LMIC cohorts often exceed 15%-20%, despite comparable operative intent [23,27,30,37].

Studies from resource-limited settings repeatedly identify late presentation as a defining feature, with many patients arriving after the onset of diffuse peritonitis, septic physiology, or hemodynamic compromise [22,37,38]. These clinical patterns are often compounded by diagnostic delays and limited access to intensive care support, which together contribute to higher postoperative morbidity and sepsis-related mortality [39-41]. Importantly, these differences appear to reflect broader health system constraints rather than intrinsic differences in surgical decision-making or technical expertise [22,44].

Variability in perioperative practice further illustrates these systemic influences. Laparoscopic repair is more frequently reported in HIC series, whereas open surgery remains predominant in LMIC cohorts, largely driven by resource availability and patient condition at presentation rather than by evidence of inferior outcomes with minimally invasive techniques [27,36,45]. Similar disparities are observed in antimicrobial management, where prolonged antibiotic courses are more commonly reported in settings characterized by delayed source control and greater contamination burden, reflecting pragmatic adaptation rather than guideline deviation [39,50-54].

Contextual Factors and Subgroups

Elderly patients and those with diabetes, cardiovascular disease, or HIV consistently show higher complication rates and longer LOS, arguing for proactive ICU involvement and organ-support strategies [26,29,33]. National data suggest that antithrombotic therapy does not substantially increase intraoperative blood loss in generalized peritonitis; therefore, it should not delay urgent source control when indicated [37]. As laparoscopic programs expand, explicit conversion thresholds and team training are essential to avoid time loss in unstable or grossly contaminated cases [28,32,46].

Extent of resection and technique selection

A key finding of our review is the persistent controversy over the role of gastric resection vs. patch repair. Zhu et al. synthesized more than 2,300 cases, demonstrating significantly lower mortality and complication rates with omental patch repair than gastric resection [30]. Gastric resection remains reserved for malignant, recurrent, or unusually large perforations [29,47]. Our pooled synthesis corroborates this trend, supporting omental patch repair as the standard in most cases, with selective resection reserved for carefully selected circumstances. Alternative techniques, including falciform ligament repair [48] or novel adjuncts such as fibrin glue [33], have been explored, but evidence remains insufficient to support widespread adoption.

Influence of demographics and comorbidities

Several studies underscored the impact of patient-related factors on outcomes. Elderly patients, those with immunosuppression, and those with significant comorbidities such as diabetes or cardiovascular disease were consistently at higher risk of mortality [24,26,28,34]. In China, Zheng et al. demonstrated a markedly higher mortality among elderly patients with perforated ulcers, further exacerbated in rural hospitals [29]. In Malawi, the presence of HIV infection was associated with higher complication rates, though not independently with mortality [32]. These findings highlight the need for tailored perioperative management strategies in vulnerable subgroups, including careful hemodynamic support, early nutritional interventions, and consideration of ICU-level care where available.

**Figure 2:**
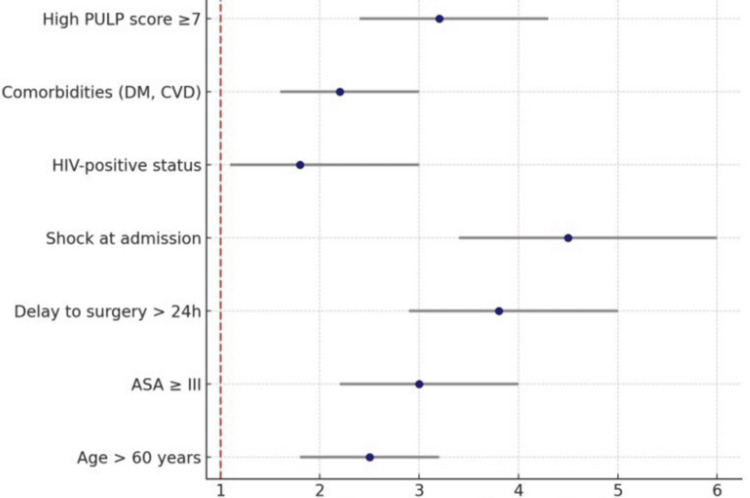
Predictors of poor outcome in generalized peritonitis due to gastric perforation, including delayed presentation, shock at admission, advanced age, and ASA ≥III PULP: peptic ulcer perforation; DM: diabetes mellitus; CVD: cardiovascular disease; ASA: American Society of Anesthesiologists Image credit: This is an original image created by the author Andreina Rosario Rosario

The outcomes of gastric perforation complicated by generalized peritonitis are not solely determined by the surgical approach but are profoundly influenced by patient- and disease-related factors. Our review confirms that advanced age and physiological reserve play a decisive role, with patients over 60 years consistently demonstrating higher mortality and complication rates [42]. Similarly, an ASA score of III or greater has been shown to be an independent predictor of adverse outcomes, reflecting the impact of comorbidities on perioperative resilience [43]. Delayed presentation, particularly beyond 24 hours after perforation, is among the strongest determinants of poor prognosis, a finding repeatedly documented in both high- and low-resource settings [44]. Hemodynamic instability at admission, especially shock, correlates with a marked rise in sepsis-related mortality [45]. Additional contributors include HIV infection, which is more relevant in sub-Saharan cohorts, and the presence of chronic comorbidities such as diabetes and cardiovascular disease that compromise postoperative recovery [46]. Finally, risk stratification tools, such as the PULP score, have demonstrated utility in quantifying these factors, thereby aiding early decision-making and prognostic counseling [47]. Collectively, these predictors underscore the importance of rapid triage, aggressive resuscitation, and context-specific perioperative planning to mitigate risk. Figure 2 presents a conceptual summary of clinical and system-level predictors consistently associated with adverse outcomes following perforated peptic ulcer surgery, rather than pooled meta-analytic effect sizes. Predictors were selected based on recurring associations reported across large observational cohorts and meta-analyses, including high PULP score (≥7), advanced age (>60 years), ASA physical status ≥III, comorbidities (diabetes mellitus, cardiovascular disease), HIV-positive status, shock at admission (defined according to Sepsis-3 criteria), and surgical delay exceeding 24 hours.

Resource-limited settings and global disparities

Perhaps the most striking observation is the consistent gap in outcomes between HICs and LMICs. Mortality in well-resourced centers often hovers around 5%-10%, whereas in sub-Saharan Africa and South Asia, figures as high as 20% remain common [23,30,36]. The Ethiopian systematic review by Endeshaw et al. [23] and the retrospective study from Jigjiga town by Burale et al. [31] both emphasize the roles of delayed presentation, limited access to diagnostic imaging, and scarce intensive care capacity. These data parallel findings from broader global audits of emergency surgery, which repeatedly highlight disparities in perioperative mortality across income strata [51-54]. The implications are profound: without structural improvements in health systems, including referral pathways, surgical training, and perioperative care, mortality gaps will persist despite advances in surgical technique [55-59].

Emerging technologies and future directions

Several studies explored adjunctive strategies to improve outcomes. Artificial intelligence applications, although still largely theoretical, hold potential in risk stratification and decision support, as discussed by Umar et al. [58]. Similarly, enhanced recovery after surgery (ERAS) protocols have shown early promise in emergency laparotomy, though robust data in peritonitis are limited [53,56]. Beyond technical innovations, system-level interventions such as standardized sepsis bundles and regional surgical networks may yield the greatest gains, particularly in LMICs.

Strengths and limitations

This review has several strengths, including its comprehensive scope and the integration of data from diverse geographic and socioeconomic settings, which enhances the external relevance of the findings. By synthesizing evidence from both high- and low-resource contexts, the review captures real-world practice patterns across a broad clinical spectrum.

However, important limitations must be acknowledged. Much of the available evidence derives from nonrandomized and retrospective studies, including case series, which introduces inherent selection bias and limits generalizability. Considerable heterogeneity exists in study design, surgical techniques, perioperative management, and outcome reporting across regions and institutions. Incomplete or missing data in retrospective cohorts further complicate interpretation. In addition, the scarcity of RCTs, limited stratification by etiology, surgical approach, or sepsis severity, and the lack of consistent long-term follow-up restrict conclusions regarding recurrence and functional outcomes [48-53]. Despite these constraints, efforts were made to prioritize higher quality studies and to perform a rigorous synthesis of the available data.

Implications for clinical practice and policy

The collective evidence affirms that timely diagnosis, rapid surgical intervention, and aggressive perioperative care remain the cornerstones of survival in gastric perforation-induced peritonitis. While laparoscopic repair offers advantages when feasible, omental patch repair remains the global standard of care. Bridging disparities between high- and low-resource settings will require not only surgical training and equipment but also systemic investment in emergency care infrastructure, referral systems, and perioperative critical care. Finally, emerging technologies such as AI-driven prognostic models and ERAS protocols offer avenues for innovation, although their impact must be validated across diverse contexts.

## Conclusions

Generalized peritonitis secondary to gastric perforation remains one of the most lethal abdominal emergencies worldwide. Despite advances in surgical technique, mortality is still driven by delayed presentation, uncontrolled sepsis, and limited access to critical care. Laparoscopic repair reduces morbidity and length of stay, yet the decisive factor for survival continues to be timely intervention rather than the operative approach itself.

Standardized sepsis protocols, early recognition, and robust perioperative resuscitation represent the cornerstone of improved outcomes. Omental patch repair remains the most effective first-line strategy in most cases, while more extensive resections should be reserved for selected, complex scenarios. The growing use of validated risk scores provides valuable guidance for triage and operative planning but must be adapted to different health-care settings.

Ultimately, narrowing the outcome gap between high-income and resource-constrained environments requires not only technical refinement but also system-wide investments in rapid referral pathways, surgical training, and critical care capacity. Generalized peritonitis due to gastric perforation is a modifiable global health challenge its burden can be substantially reduced through early diagnosis, decisive surgery, and equitable access to care.
